# Analysis of *PPARGC1B*, *RUNX3* and *TBKBP1* Polymorphisms in Chinese Han Patients with Ankylosing Spondylitis: A Case-Control Study

**DOI:** 10.1371/journal.pone.0061527

**Published:** 2013-04-18

**Authors:** Zijian Lian, Wei Chai, Lewis L. Shi, Chao Chen, Jingyi Liu, Yan Wang

**Affiliations:** 1 Department of Orthopaedics, Chinese People’s Liberation Army General Hospital, Beijing, China; 2 Medical School of Nankai University, Tianjin, China; 3 Department of Orthopaedics, Tianjin Hospital, Tianjin, China; 4 Department of Orthopaedics, University of Chicago Hospital, Maryland Avenue, Chicago, Illinois, United States of America; Tor Vergata University of Rome, Italy

## Abstract

**Background:**

Susceptibility to and severity of ankylosing spondylitis (AS) are largely genetically determined. *PPARGC1B*, *RUNX3* and *TBKBP1* have recently been found to be associated with AS in patients of western European descent. Our purpose is to examine the influence of *PPARGC1B*, *RUNX3* and *TBKBP1* polymorphisms on the susceptibility to and the severity of ankylosing spondylitis in Chinese ethnic majority Han population.

**Methods:**

Blood samples are drawn from 396 AS patients and 404 unrelated healthy controls. All the patients and the controls are Han Chinese and the patients are HLA-B27 positive. The AS patients are classified based on the severity of the disease. Twelve tag single nucleotide polymorphisms (tagSNPs) in *PPARGC1B*, *RUNX3* and *TBKBP1* are selected and genotyped. Frequencies of different genotypes and alleles are analyzed among the different severity AS patients and the controls.

**Results:**

After Bonferroni correction, the rs7379457 SNP in *PPARGC1B* shows significant difference when comparing all AS patients to controls (p = 0.005). This SNP also shows significant difference when comparing normal AS patients to controls (p = 0.002). The rs1395621 SNP in *RUNX3* shows significant difference when comparing severe AS patients to controls (p = 0.007). The rs9438876 SNP in *RUNX3* shows significant difference when comparing normal AS patients to controls (p = 0.007). The rs8070463 SNP in *TBKBP1* shows significant difference in genotype distribution when comparing severe AS patients to controls (p = 0.003).

**Conclusions:**

The rs7379457 SNP in *PPARGC1B* is related to susceptibility to AS in Chinese Han population. The rs7379457 SNP in *PPARGC1B,* the rs1395621 and rs9438876 SNPs in *RUNX3,* and the rs8070463 SNP in *TBKBP1* are related to the severity of AS in Chinese Han population.

## Introduction

Ankylosing spondylitis (AS) is a chronic inflammatory disorder characterized by inflammation in the spine and sacroiliac joints causing initial bone and joint erosion and subsequent ankylosis [1]. Most patients develop first symptoms of AS younger than 30 years of age [2]. Significant radiographic progression occurs in the first 10 years of disease, and more recent studies have shown that structural damage at initial presentation is the best predictor of further damage [3]–[5].

AS patients’ disease severity is largely genetically determined [6]. We aim to identify patients with susceptibility to AS before the appearance of significant deformities and disabilities, so we can intervene earlier. In genome-wide association studies (GWAS), rs11959820 in *PPARGC1B*, rs11249215 in *RUNX3*, and rs8070463 in *TBKBP1* are related to AS susceptibility in patients of western European descent [7]–[9]. These findings must be replicated and refined in different populations. Interpreting GWAS results at a gene-specific level is an important step towards understanding the molecular processes that lead to the disease [10].

We hypothesize that the *PPARGC1B, RUNX3*, and *TBKBP1* are related to AS in the Chinese ethnic majority Han population. Additionally, some single nucleotide polymorphisms (SNPs) of these genes may predict the severity of AS.

## Methods

### Study Population

In this work, 396 AS patients are recruited, along with 404 unrelated healthy controls who are age and sex-matched. All patients and controls are Han Chinese. All AS patients are HLA-B27 positive. All AS patients are treated by non-steroidal anti-inflammatory drug routinely; no other treatments are used for patients. Among the AS patients, there are 354 males (89.4%) and 42 females (10.6%); the average age is 29.6 years (range 16 to 60 years) (Table 1). Among the controls, there are 364 males (90.1%) and 40 females (9.9%); the average age is 30.0 years (range 16 to 60 years). Neither sex nor age distributions show significant differences between AS and control patients (p = 0.742, 0.518 respectively). The average duration since AS diagnosis is 11.5 years (range 8 to 18 years). The diagnosis of AS has been made by experienced rheumatologists; all diagnoses satisfy the modified New York criteria [11]. Subjects with inflammatory bowel disease, psoriasis, rheumatoid arthritis, or other autoimmune diseases are excluded from both the AS and the control groups.

**Table 1:** Demographic data of AS patients and controls.

Sex		Cases (396)	Controls (404)	p-value
	male	354 (89.4%)	364 (90.1%)	0.742
	female	42(10.6%)	40 (9.9%)	
Age		29.6±8.5	30.0±9.4	0.518
Duration of diagnosis		11.5±2.1	N/A	
BASFI		3.97±1.49	N/A	
BASDAI		3.95±1.05	N/A	
mSASSS		12.6±13.4	N/A	

There is no significant difference in age and sex-distribution between AS patients and controls. Numerical values presented as mean±standard deviation. BASFI : Bath ankylosing spondylitis function index. BASDAI: Bath ankylosing spondylitis disease activity index. mSASSS: modified Stokes ankylosing spondylitis Spine Score.

### Basic Data Acquisition

The Bath AS function index (BASFI) and Bath AS disease activity index (BASDAI) are administered to the patients using questionnaires; these indices are the most widely used tools for the assessment of AS functional status and disease activity [12] [13]. The modified Stokes AS Spine Score (mSASSS) is a validated scoring system for quantification of chronic spinal changes [14]. Standard anteroposterior and lateral radiographs of the cervical and lumbar spine are obtained for each subject, and the lateral view is used to derive a mSASSS score for each patient [15] [16]. Three of the authors separately assigned the mSASSS scores, and the average is used.

### Severity Classification

There is a lack of consensus on how to classify AS severity [17]. In this work, we define severe subtype of AS as the disease form in those patients who require surgery within first ten years of diagnosis. The indications of surgery include inability to stand upright, inability to look straight ahead, and compression of the viscera due to kyphosis that manifests as pain [18]. Patients with the normal type of AS exhibit inflammation of sacroiliac joints, but their spine and other joints are relatively spared; these patients require only medical treatment. By this definition, 82 AS patients have the severe subtype, and 314 AS patients have the normal subtype. Clinical features comparing severe AS and normal AS are shown in Table 2.

**Table 2:** Clinical features comparing severe AS and normal AS.

Sex		severe AS (82)	normal AS (314)	p value
	male	76(92.7%)	276(87.9%)	0.220
	female	6(7.3%)	38(12.1%)	
Age		31.5±9.2	29.0±8.3	0.097
Duration of diagnosis		11.2±3.0	11.6±1.8	0.290
BASFI		6.03±2.06	3.43±0.55	<0.001
BASDAI		5.49±1.10	3.55±0.55	<0.001
mSASSS		37.1±13.3	6.21±1.06	<0.001

There is no difference between severe AS patients and normal patients in age and sex distribution; however, the BASFI, BASDAI and mSASSS are higher in severe AS patients.

### SNPs Selection

**Figure 1:**
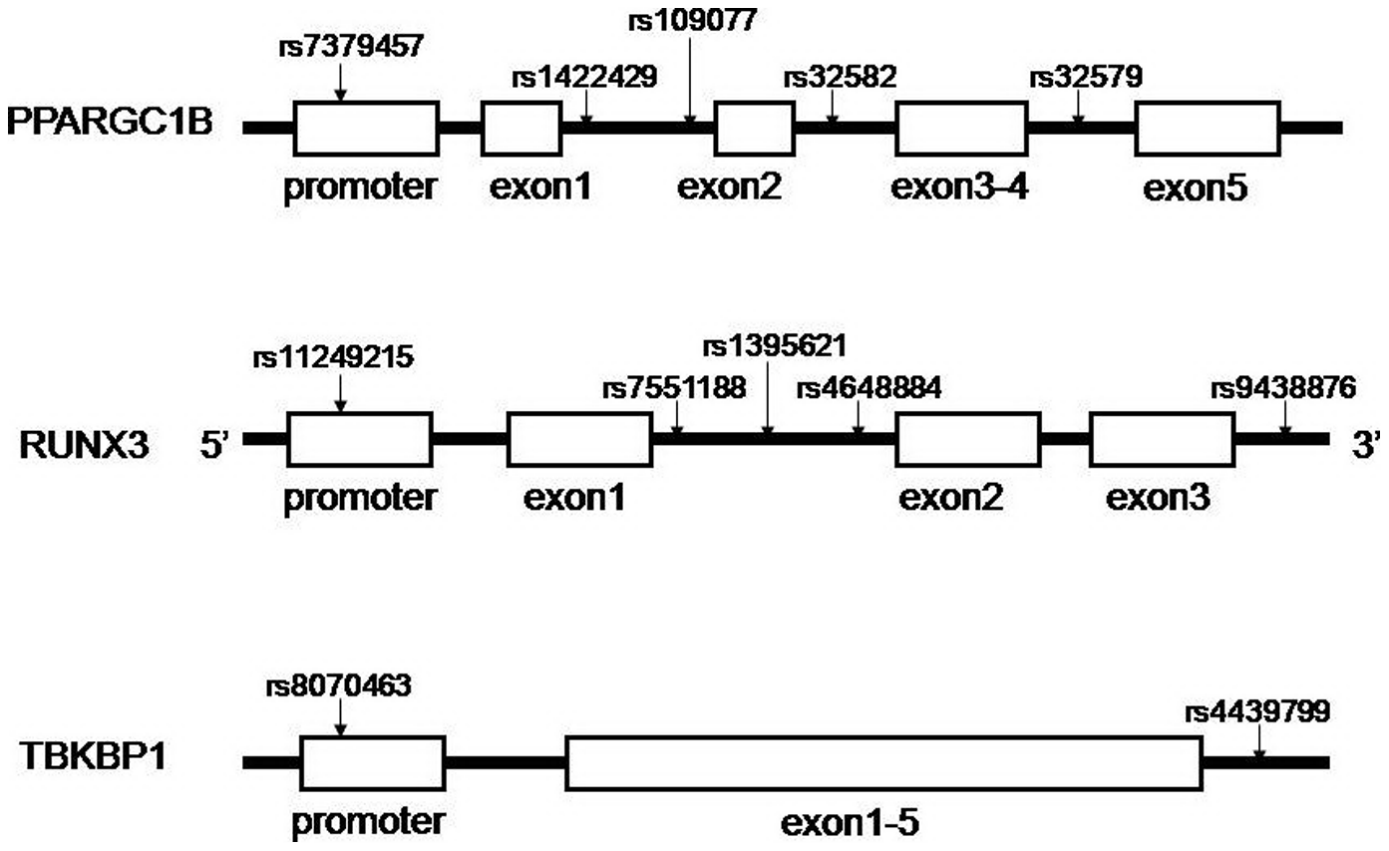
Positions of each selected tagSNP on the genes. The SNP rs7379457 is in the promoter of PPARGC1B. The SNP rs11249215 is in the promoter of RUNX3. The SNP rs8070463 is in the promoter of TBKBP1. Other SNPs are all in introns.

The SNPs in this study include five in *PPARGC1B*, five in *RUNX3* and two in *TBKBP1*. These three genes localize to chromosome 5, 1, and 17, respectively. The selected SNPs serve as multi-marker tagging algorithm with criteria of r^2^ more than 0.8 and for all SNPs with minor allele frequency more than 5% from the Han Chinese in Beijing population in the HapMap database. Haploview 4.2 software (Broad Institute, Cambridge, Massachusetts, USA) is used to select the tagSNPs. Figure 1 shows the positions of each tagSNP. The rs7379457 SNP is located in the promoter of *PPARGC1B*. The rs11249215 SNP is located in the promoter of *RUNX3*. The rs8070463 SNP is located in the promoter of *TBKBP1*. Other SNPs are located in the introns. Most SNPs in *TBKBP1* are in high linkage disequilibrium (LD), hence only two tagSNPs are selected.

### DNA Extraction and Genotyping Analysis

DNA is isolated from 2 ml whole blood samples using AxyPrep Blood Genomic DNA Miniprep kit (Axygen Biosciences, Union City, CA, USA). Detection of the SNPs is performed by MassARRAY system (Sequenom, San Diego, CA, USA). The chip-based matrix-assisted laser desorption ionization time-of-flight (MALDI-TOF) mass spectrometry technology is used in this procedure [19]. Most of the SNPs are successfully genotyped. The rs109077 SNP is 98.5% genotyped in case group and 97.5% in control group. The rs4648884 SNP is 98.5% genotyped in case group. The rs9438876 is 90.9% genotyped in case group and 95.5% in control group. In the other SNPs, more than 99.5% is genotyped in both case and control groups.

### Statistical Analysis

The Hardy-Weinberg equilibrium is tested for all 12 tagSNPs. The Pearson’s chi-squared test and independent-samples t-test are used to compare the differences in age and sex between cases and controls. Comparisons of the distributions of the genotype, allele and haplotype frequencies are performed using the Pearson’s chi-squared test. Especially, in the rs7379457 SNP the TT genotype is rare. Therefore, we use Fisher’s exact test. Binary logistic regression analysis is used to adjust for age and sex. After Bonferroni correction, p-value less than 0.01 is considered significant. The last genotype of each SNP is the major genotype and the last allele is the major allele. The relative risks associated with the major genotypes or major alleles are estimated as an odds ratio (OR) with a 95% confidence interval (CI). The p-values of genotypes indicated in the result tables are used to estimate the significance of the distribution of genotype between cases and controls. All three genotypes of each SNP are compared, P-value for individual genotypes are shown only if significant at 0.05 level (Table 3, details are shown in Table S1, Table S2 and Table S3). We compared the severe AS group to the entirety of the control group and then normal AS group to the entirety control group. Pearson’s chi-squared test is used to compare the constructed haplotypes. The SNPs which show significant differences between AS patients and controls are considered to be related to susceptibility to AS. The SNPs which show significant differences between severe AS patients and controls but no differences between normal AS patients and controls are considered related to severity of AS. Moreover, SNPs show significant differences between normal AS patients and controls but no differences between severe AS patients and controls are considered related to severity of AS. Statistical analyses are carried out with SPSS v.17.0 software package (IBM, Armonk, New York, USA).

**Table 3:** Positive SNPs in *PPARGC1B RUNX3* and *TBKBP1* which are related to susceptibility to AS or severity of AS comparing all AS patients, severe AS patients and normal AS patients to the controls.

	SNP		All AS subjects cases/controls			Severe AS subjects cases/controls			Normal AS subjects cases/controls		
			frequencies	OR(95% CI)^c^	p	frequencies	OR(95% CI)	p	frequencies	OR(95% CI)	p
***PPARGC1B***	**rs7379457**	All^a^			N/A			N/A			N/A
	Genotype	TT	8/0	1.024(1.007∼1.041)	**0.005** *	0/0	N/A	N/A	8/0	1.029(1.009∼1.050)	**0.002** *
		CT	50/72	0.671(0.453∼0.993)	0.046#	18/72	1.292(0.714∼2.339)		32/72	0.538(0.344∼0.841)	**0.006** *
		CC	336/330	1^b^		64/330	1		272/330	1	
	Allele	T	66/72	0.929(0.655∼1.318)		18/72	1.253(0.726∼2.164)		48/72	0.847(0.579∼1.240)	
		C	722/732	1		146/732	1		576/732	1	
***RUNX3***	**rs1395621**	All			0.041#			**0.008** *			0.147
	Genotype	AA	58/66	0.730(0.477∼1.117)		6/66	0.408(0.178∼0.934)	**0.007** *	52/66	0.834(0.534∼1.303)	
		AG	189/220	0.689(0.504∼0.943)	0.015#	39/220	0.538(0.321∼0.903)	0.025#	150/220	0.736(0.527∼1.028)	
		GG	149/118	1		37/118	1		112/118	1	
	Allele	A	305/352	0.811(0.665∼0.991)	0.040#	51/352	0.585(0.408∼0.837)	**0.003** *	254/352	0.880(0.712∼1.087)	
		G	487/456	1		113/456	1		374/456	1	
	**rs9438876**	All			0.022#			0.716			**0.004** *
	Genotype	GG	43/30	1.347(0.807∼2.251)		4/30	0.556(0.180∼1.713)		39/30	1.590(0.937∼2.699)	
		AG	129/172	0.743(0.547∼1.008)	0.047#	32/172	0.905(0.538∼1.523)		97/172	0.701(0.504∼0.974)	0.028#
		AA	188/184	1	0.038#	38/184	1		150/184	1	**0.007** *
	Allele	G	215/232	0.991(0.794∼1.237)		40/232	0.862(0.581∼1.278)		175/232	1.026(0.811∼1.298)	
		A	505/540	1		108/540	1		397/540	1	
***TBKBP1***	**rs8070463**	All			0.187			0.014#			0.415
	Genotype	CC	72/94	0.788(0.531∼1.170)		8/94	0.400(0.186∼0.861)	**0.003** *	64/94	0.928(0.611∼1.411)	
		CT	198/184	1.084(0.786∼1.494)		40/184	0.815(0.483∼1.376)		158/184	1.170(0.828∼1.654)	
		TT	124/124	1		34/124	1		90/124	1	
	Allele	C	342/372	0.890(0.731∼1.085)		56/372	0.602(0.424∼0.856)	**0.004** *	286/372	0.983(0.797∼1.212)	
		T	446/432	1		108/432	1		338/432	1	

a “All” means the p value that we compare all the three genotype using 3×2 chi squared method. P-value for individual genotypes are shown only if significant at 0.05 level.

b The last lines of genotypes or alleles are the major genotypes or the major alleles. The other genotypes or alleles are compared to them. The relative risk associated with major genotypes and major alleles is estimated as an odds ratio (OR) with a 95% confidence interval (CI).

c OR (95% CI) are adjusted by age and sex using multiple regression analysis.

# indicates p-value is less than 0.05 but cannot pass Bonferroni correction which shows marginal significant difference.

* indicates p-value is less than 0.01 which shows significant difference after Bonferroni correction. The details of all the 12 SNPs are summarized in Table S1 (*PPARGC1B*) Table S2 (*RUNX3*) and Table S3 (*TBKBP1*).

### Ethics Statement

The blood samples of both AS patients and controls used in this study are part of samples taken for diagnostic tests. During the collection and use of DNA samples, clinical data guidelines, regulations of the local Ethics Committee and the Helsinki Declaration in 1975 are followed. Written informed consents were obtained from all the patients and subjects (or their parents in the case of two patients less than 18 years old). The study procedure is approved by our Institutional Review Board.

## Results

### Clinical Features

The BASFI, BASDAI and mSASSS for the AS patients are recorded in Table 1. Among the 396 AS patients, the mean BASFI is 3.97±1.49 (mean±standard deviation). The mean BASDAI is 3.95±1.05. The mean mSASSS is 12.6±13.4. When comparing the severe AS and the normal AS patient groups, there is no significant difference in sex, age, and disease duration (p-value = 0.220, 0.097, 0.290 respectively; Table 2). The BASFI is higher in severe AS group (6.03±2.06) than normal AS group (3.43±0.55) (p-value<0.001), reflecting poorer function of patients in the severe AS group. The BASDAI is similarly higher in the severe AS group (5.49±1.10) than normal AS group (3.55±0.55) (p-value<0.001), reflecting higher disease activity. The pattern holds for mSASSS (37.1±13.3 versus 6.21±1.06, p-value<0.001), signifying more radiographic changes in the severe AS patients.

### Genotype and Allele

We genotyped 12 SNPs, the detailed results are summarized in Table S1, Table S2, and Table S3. The genotype frequencies of these 12 tagSNPs are in Hardy-Weinberg equilibrium in all groups. Four of the 12 SNPs show significant difference in disease diagnosis and severity (Table 3). The genotype frequencies of these 12 tagSNPs are in Hardy-Weinberg equilibrium in all groups. After Bonferroni correction, the rs7379457 SNP in *PPARGC1B* shows significant difference when comparing all AS patients to controls, with TT genotype higher in AS than in controls (p = 0.005). This SNP also shows significant difference when comparing normal AS patients to controls, with TT genotype higher in normal AS than in controls (p = 0.002) and CC genotype higher in normal AS than in controls (p = 0.006). The rs1395621 SNP in *RUNX3* shows significant difference when comparing severe AS patients to controls, with AA genotype lower in severe AS patients than in controls (p = 0.007) and A allele is lower in severe AS patients than in controls (p = 0.003). The rs9438876 SNP in this gene shows significant difference when comparing normal AS patients to controls, with GG genotype lower in normal AS patients than in controls (p = 0.007). The rs8070463 SNP in *TBKBP1* shows significant difference in genotype distribution when comparing severe AS patients to controls, with CC genotype lower in severe AS patients than in controls (p = 0.003). This SNP also shows significant difference in C allele distribution when comparing severe AS patients to controls, with C allele lower in severe AS patients than in controls (p = 0.004).

### Haplotype

**Figure 2:**
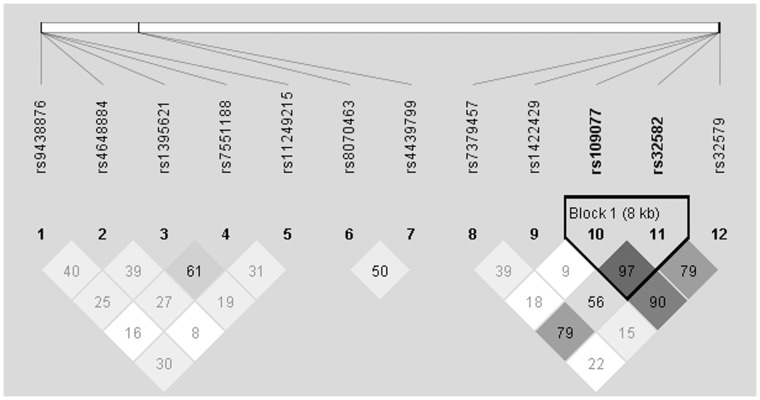
Linkage disequilibrium (LD) map comparing All AS patients and controls. Darker color indicates higher linkage disequilibrium (LD), lighter color indicates less LD. Numbers in the squares indicate correlation coefficient (R^2^) value. The left part of the picture contains 5 SNPs (from rs9438876 to rs11249215). They are from *RUNX3*. The middle part of the picture contains 2 SNPs (rs8070463 and rs4439799). They are from *TBKBP1*. The right part of the picture contains 4 SNPs (from rs7379457 to rs32579). They are from *PPARGC1B.* Haplotypes are constructed from the darker blocks (high linkage disequilibrium). They are TG, GT and GG.

LD maps of the 12 tagSNPs of *PPARGC1B*, *TBKBP1* and *RUNX3* comparing all AS patients, severe AS patients, and normal AS patients to controls subjects are shown in Figure 2, Figure S1 and Figure S2, respectively. These figures have only a little difference, only Figure 2 is shown in the text comparing all AS patients to controls. Figure S1 and Figure S2 are shown in the supporting information comparing severe patients and normal patients to controls separately. Most of the SNPs are not in high linkage disequilibrium, with exception of one block in *RUNX3*. Analyses of constructed haplotypes for this block comparing all AS patients, severe AS patients, and normal AS patients to controls subjects are shown in Table 4. None of them is associated with the susceptibility to AS or severity of AS.

**Table 4:** Haplotype analysis in block 1.

	rs109077	rs32582	Case Ratio	Control Ratio	Chi Square	p value
All AS patients vs. controls	T	G	529∶263	536∶272	0.037	0.847
	G	T	136∶656	141∶667	0.022	0.883
	G	G	123∶669	130∶678	0.094	0.759
Severe AS patients vs. controls	T	G	114∶50	537∶271	0.574	0.449
	G	T	28∶136	142∶666	0.024	0.878
	G	G	22∶142	130∶678	0.739	0.390
Normal AS patients vs. controls	T	G	415∶213	536∶272	0.010	0.920
	G	T	108∶520	141∶667	0.016	0.900
	G	G	101∶527	130∶678	1.04E-5	0.997

Haplotypes are constructed. Case ratio means in the case group, the frequency of this kind of haplotype vs. other kinds of haplotype; control ratio means in the control group, the frequency of this kind of haplotype vs. other kinds of haplotype. None of the constructed haplotype has significant difference between cases and controls.

## Discussion

The pathogenesis of AS remains poorly understood. However, genetic factors play a significant role [20]. Changes in the spine involve syndesmophytes forming bony ankylosis of adjacent vertebrae or ankylosis of small vertebral joints. Some classification systems exist based on clinical and radiographic criteria [21] [22]. Most of our patients present with mild symptoms and do well with chronic medical treatments; however, some patients have more severe manifestations that require surgery within the first ten years of diagnosis. We use this criterion to define severe and normal AS disease subtypes. We aim to find genetic markers for susceptibility to AS in general, as well as to the severe form of AS. This would provide powerful tools to clinicians and researchers to confirm the diagnosis of AS and predict for the development of severe form of AS. The ability to classify AS based on allelic differences would also have significant new therapies implications [23].

The human *PPARGC1B* gene (peroxisome proliferator-activated receptor-gamma coactivator 1 beta), encoding PGC-1β, localizes to chromosome 5q32, a region that shows linkage to type 2 diabetes [24]. Wirtenberger and colleagues found PGC-1β to be associated with familial breast cancer [25]. The mechanism that *PPARGC1B* relates to these diseases is still being investigated. Numerous publications indicate *PPARGC1B* plays a critical role in regulating multiple aspects of energy metabolism including mitochondrial biogenesis, thermogenesis, gluconeogenesis, and fatty acid β-oxidation [26]–[30]. Oxidative phosphorylation (OXPHOS) dysfunction plays a critical pathogenic role in several human diseases [31]. Sarika found that PGC-1β over-expression can lead to a marked improvement in OXPHOS defects caused by mutations in mitochondrial DNA (mtDNA) or nuclear DNA (nDNA) [32]. Kiyo-aki found PGC-1β accelerated osteoclastic bone resorption through the coordination of mitochondrial biogenesis and the cell-differentiation program [33]. Our data shows that rs7379457 SNP which localizes to the promoter of *PPARGC1B* is related to the susceptibility to AS and severity of AS. As a result, the functions of osteoclasts are inhibited due to decreased oxidative phosphorylation. With the imbalance between osteoblasts and osteoclasts, ossification predominates and thus leads to osteophyte formation and even ankylosis.

The *RUNX3* gene (Runt-related transcription factor 3) encodes for RUNX3, and it localizes to chromosome 1p36.11. RUNX3 is a downstream target of the transforming growth factor-β (TGF-β) pathway, which is considered a tumor suppressor pathway, as components are frequently altered in cancers, especially those of the gastrointestinal tract [34]. *RUNX3* is inactivated in gastric cancer by hemizygous deletion, promoter hypermethylation, histone modification, and protein mislocalization, suggesting a tumor-suppressive role of RUNX3 in this malignancy [35]–[37]. Since the discovery of the potential role of RUNX3 in the initiation and the progression of gastric cancer, RUNX3 has been found to be involved in the development of a variety of cancers, including colon, liver, lung and breast cancer [38]–[41]. *RUNX3* knockout mice spontaneously develop inflammatory bowel disease characterized by leukocyte infiltration, mucosal hyperplasia, formation of lymphoid clusters, and increased production of IgA. RUNX3 belongs to the runt domain family of transcription factors, which are key regulators of lineage-specific gene expression and more recently are found to be linked to human autoimmunity [42]. When RUNX3 is suppressed in human T cells, either through gene inactivation or with small interference RNA, Foxp3 expression is reduced, which in turn disrupts the recognition of regulatory T cells. As with other autoimmune diseases, AS patients exhibit an imbalance of CCR4+CCR6+ helper T cells and regulatory T cells [43] [44]. RUNX3 is highly expressed in dendritic cells (DC), where it functions as a component of the transforming growth factor (TGF-β) signaling cascade [45]. It is obvious that RUNX3 is not only a tumor suppressor but also plays an important role in autoimmune diseases and inflammations. In our data, the rs1395621 and rs9438876 SNP show significant relationship to severity of AS. We conclude the RUNX3 can influence the AS severity due to its effect on the inflammatory process.

TBKBP1 (tumor necrosis factor family member-associated NF-κB activator binding kinase 1 binding protein) is an adaptor protein that binds to TBK1, also known as sintbad. The precise function of TBKBP1 in the process of TBK1 activation has not been defined fully, but there is evidence that the adaptor proteins link the kinases to the upstream signaling pathways, possibly by its interaction with TRAF3 [46]. Ken performed bone-marrow transfer experiments which revealed that TBK1-mediated signaling in hematopoietic cells is critical for the induction of antigen specific B and CD4+ T cells, whereas in non-hematopoietic cells TBK1 is required for CD8+ T-cell induction. These data suggest that TBK1 is a key signaling molecule for DNA-vaccine-induced immunogenicity, in addition to being part of the classic NF-κB inflammatory pathway [47]. Acute or chronic inflammation is an important feature of AS, with degree of inflammation correlated to severity of disease [48]. Appropriately, our data shows that rs8070463 SNP of *TBKBP1* is related to the severity of AS.

In conclusion, *PPARGC1B* is associated with the susceptibility to AS; *PPARGC1B*, *RUNX3* and *TBKBP1* are associated with the severity of AS in the Chinese Han population. These findings support the GWAS results that these three genes are related to AS. Our findings can provide context for better understanding of the genetic and molecular pathogenesis of AS. The specific SNPs in these genes can be used to guide genetic analysis and counseling, medical and surgical treatment options, and ultimate prognosis. Further studies are needed to elucidate the molecular roles these genes play in AS.

## Supporting Information

Figure S1 **Linkage disequilibrium map comparing severe AS patients and controls.** The distribution and position of SNPs are the same as Figure 2. Haplotypes are constructed from the darker blocks (high linkage disequilibrium). They are TG, GT and GG.
(TIF)

Figure S2 **Linkage disequilibrium map comparing normal AS patients and controls.** The distribution and position of SNPs are the same as Figure 2. Haplotypes are constructed from the darker blocks (high linkage disequilibrium). They are TG, GT and GG.
(TIF)

Table S1 Genotype and allele frequencies of *PPARGC1B* SNPs among all AS patients, severe AS patients, normal AS patients versus controls. SNPs in *PPARGC1B* are compared between all AS patients, severe AS patients, and normal AS patients versus the control subjects. P-value for each SNP is shown, and p-value for individual genotypes are shown only if significant at 0.05 level. # indicates p-value is less than 0.05 but cannot pass Bonferroni correction which shows marginal significant difference. *indicates p-value is less than 0.01 which shows significant difference after Bonferroni correction. OR and 95% CI are adjusted by age and sex. The rs7379457 SNP shows significant difference when comparing all AS patients to controls, TT genotype carrier frequency is higher than controls (p = 0.005*). This SNP also shows significant difference in comparing normal AS patients to controls, TT genotype carrier frequency is higher than controls (p = 0.002*); CC genotype carrier frequency is lower than controls (p = 0.006*).
(DOCX)

Table S2 Genotype and allele frequencies of *RUNX3* SNPs among all AS patients, severe AS patients, normal AS patients versus controls. SNPs in *RUNX3* are compared between all AS patients, severe AS patients, and normal AS patients versus the control subjects. # indicates p-value is less than 0.05 but cannot pass Bonferroni correction which shows marginal significant difference. *indicates p-value is less than 0.01 which shows significant difference after Bonferroni correction. After Bonferroni correction, the rs1395621 SNP shows significant difference when comparing severe AS patients to controls, AA genotype carrier frequency is lower than controls (p = 0.007*) and A allele carrier frequency is lower than controls (p = 0.003*). The rs9438876 SNP shows significant difference when comparing normal AS patients to controls, the AA genotype is lower than controls (p = 0.007*).
(DOCX)

Table S3 Genotype and allele frequencies of *TBKBP1* SNPs among all AS patients, severe AS patients, normal AS patients versus controls. SNPs in *TBKBP1* are compared between all AS patients, severe AS patients, and normal AS patients versus the control subjects. # indicates p-value is less than 0.05 but cannot pass Bonferroni correction which shows marginal significant difference. *indicates p-value shows significant difference after Bonferroni correction. After Bonferroni correction the rs8070463 SNP shows significant difference in genotype distribution when comparing severe AS patients to controls, CC genotype carrier frequency is lower than controls (p = 0.003*). This SNP also shows significant difference in C allele distribution when comparing severe AS patients to controls, C allele carrier frequency is lower than controls (p = 0.004*). The rs8070463 SNP is related to the severity of AS.
(DOCX)
