# Supplementary figures and images for: Analysis of PPARGC1B, RUNX3 and TBKBP1 Polymorphisms in Chinese Han Patients with Ankylosing Spondylitis: A Case-Control Study
Source: PLoS One. 2013 Apr 18;8(4):e61527. doi: 10.1371/journal.pone.0061527 (PMC3630117; doi:10.1371/journal.pone.0061527)

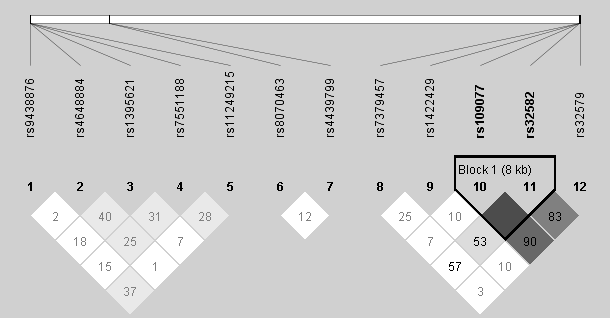

Supplement: Figure S1 — Linkage disequilibrium map comparing severe AS patients and controls. The distribution and position of SNPs are the same as Figure 2. Haplotypes are constructed from the darker blocks (high linkage disequilibrium). They are TG, GT and GG. (TIF) [file pone.0061527.s001.tif]

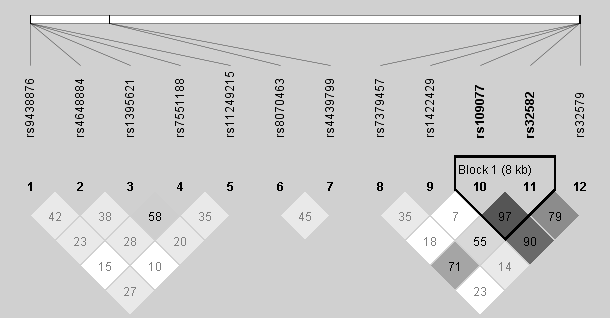

Supplement: Figure S2 — Linkage disequilibrium map comparing normal AS patients and controls. The distribution and position of SNPs are the same as Figure 2. Haplotypes are constructed from the darker blocks (high linkage disequilibrium). They are TG, GT and GG. (TIF) [file pone.0061527.s002.tif]
