# Supplementary material for: Analysis of PPARGC1B, RUNX3 and TBKBP1 Polymorphisms in Chinese Han Patients with Ankylosing Spondylitis: A Case-Control Study
Source: PLoS One. 2013 Apr 18;8(4):e61527. doi: 10.1371/journal.pone.0061527 (PMC3630117; doi:10.1371/journal.pone.0061527)
Supplement: Table S1 — Genotype and allele frequencies of PPARGC1B SNPs among all AS patients, severe AS patients, normal AS patients versus controls. SNPs in PPARGC1B are compared between all AS patients, severe AS patients, and normal AS patients versus the control subjects. P-value for each SNP is shown, and p-value for individual genotypes are shown only if significant at 0.05 level. # indicates p-value is less than 0.05 but cannot pass Bonferroni correction which shows marginal significant difference. *indicates p-value is less than 0.01 which shows significant difference after Bonferroni correction. OR and 95% CI are adjusted by age and sex. The rs7379457 SNP shows significant difference when comparing all AS patients to controls, TT genotype carrier frequency is higher than controls (p = 0.005*). This SNP also shows significant difference in comparing normal AS patients to controls, TT genotype carrier frequency is higher than controls (p = 0.002*); CC genotype carrier frequency is lower than controls (p = 0.006*). (DOCX) [file pone.0061527.s003.docx]

Table S1. Genotype and allele frequencies of *PPARGC1B* SNPs among all AS patients, severe AS patients, normal AS patients versus controls.

| SNP |  | All AS subjects cases / controls | |  | Severe AS subjects cases / controls | |  | Normal AS subjects cases / controls | |  |
| --- | --- | --- | --- | --- | --- | --- | --- | --- | --- | --- |
|  |  | frequencies | OR(95% CI) | p | frequencies | OR(95% CI) | p | frequencies | OR(95% CI) | p |
| **rs7379457** | All |  |  | N/A |  |  | N/A |  |  | N/A |
| Genotype | TT | 8/0 | 1.024(1.007~1.041) | **0.005*** | 0/0 | N/A | N/A | 8/0 | 1.029(1.009~1.050) | **0.002*** |
|  | CT | 50/72 | 0.671(0.453~0.993) | **0.046#** | 18/72 | 1.292(0.714~2.339) |  | 32/72 | 0.538(0.344~0.841) | **0.006*** |
|  | CC | 336/330 | 1 |  | 64/330 | 1 |  | 272/330 | 1 |  |
| Allele | T | 66/72 | 0.929(0.655~1.318) |  | 18/72 | 1.253(0.726~2.164) |  | 48/72 | 0.847(0.579~1.240) |  |
|  | C | 722/732 | 1 |  | 146/732 | 1 |  | 576/732 | 1 |  |
|  |  |  |  |  |  |  |  |  |  |  |
| **rs1422429** | All |  |  | 0.884 |  |  | 0.912 |  |  | 0.899 |
| Genotype | CC | 44/44 | 1.006(0.625~1.619) |  | 10/44 | 1.034(0.464~2.302) |  | 34/44 | 0.983(0.591~1.633) |  |
|  | CT | 194/206 | 0.940(0.698~1.265) |  | 40/206 | 0.934(0.558~1.563) |  | 154/206 | 0.944(0.688~1.296) |  |
|  | TT | 156/154 | 1 |  | 32/154 | 1 |  | 124/154 | 1 |  |
| Allele | C | 282/294 | 0.974(0.794~1.195) |  | 60/294 | 1.009(0.712~1.429) |  | 222/294 | 0.965(0.777~1.200) |  |
|  | T | 506/514 | 1 |  | 104/514 | 1 |  | 402/514 | 1 |  |
|  |  |  |  |  |  |  |  |  |  |  |
| **rs109077** | All |  |  | 0.817 |  |  | 0.348 |  |  | 0.940 |
| Genotype | GG | 34/38 | 0.987(0.597~1.633) |  | 4/38 | 0.503(0.168~1.508) |  | 30/38 | 1.116(0.662~1.883) |  |
|  | GT | 188/182 | 1.086(0.809~1.457) |  | 42/182 | 1.072(0.653~1.761) |  | 146/182 | 1.075(0.785~1.472) |  |
|  | TT | 168/174 | 1 |  | 36/174 | 1 |  | 132/174 | 1 |  |
| Allele | G | 256/258 | 1.004(0.813~1.239) |  | 50/258 | 0.901(0.626~1.297) |  | 206/258 | 1.032(0.825~1.291) |  |
|  | T | 524/530 | 1 |  | 114/530 | 1 |  | 410/530 | 1 |  |
|  |  |  |  |  |  |  |  |  |  |  |
| **rs32582** | All |  |  | 0.326 |  |  | 0.885 |  |  | 0.297 |
| Genotype | TT | 8/14 | 0.595(0.245~1.446) |  | 2/14 | 0.792(0.174~3.604) |  | 6/14 | 0.549(0.207~1.456) |  |
|  | GT | 124/114 | 1.139(0.839~1.547) |  | 24/114 | 0.955(0.559~1.633) |  | 100/114 | 1.167(0.844~1.614) |  |
|  | GG | 264/276 | 1 |  | 56/276 | 1 |  | 208/276 | 1 |  |
| Allele | T | 140/142 | 1.007(0.779~1.302) |  | 28/142 | 0.966(0.619~1.507) |  | 112/142 | 1.018(0.775~1.338) |  |
|  | G | 652/666 | 1 |  | 136/666 | 1 |  | 516/666 | 1 |  |
|  |  |  |  |  |  |  |  |  |  |  |
| **rs32579** | All |  |  | 0.813 |  |  | 0.085 |  |  | 0.936 |
| Genotype | AA | 38/44 | 0.844(0.525~1.359) |  | 4/44 | 0.484(0.163~1.439) |  | 34/44 | 0.922(0.563~1.510) |  |
|  | AG | 172/170 | 1.034(0.770~1.387) |  | 44/170 | 1.379(0.838~2.270) |  | 128/170 | 0.941(0.687~1.290) |  |
|  | GG | 184/188 | 1 |  | 34/188 | 1 |  | 150/188 | 1 |  |
| Allele | A | 248/258 | 0.972(0.787~1.200) |  | 52/258 | 0.983(0.685~1.409) |  | 196/258 | 0.969(0.774~1.213) |  |
|  | G | 540/546 | 1 |  | 112/546 | 1 |  | 428/546 | 1 |  |

SNPs in *PPARGC1B* are compared between all AS patients, severe AS patients, and normal AS patients versus the control subjects. P-value for each SNP is shown, and p-value for individual genotypes are shown only if significant at 0.05 level. # indicates p-value is less than 0.05 but cannot pass Bonferroni correction which shows marginal significant difference. *indicates p-value is less than 0.01 which shows significant difference after Bonferroni correction. OR and 95% CI are adjusted by age and sex. The rs7379457 SNP shows significant difference when comparing all AS patients to controls, TT genotype carrier frequency is higher than controls (p=0.005*). This SNP also shows significant difference in comparing normal AS patients to controls, TT genotype carrier frequency is higher than controls (p=0.002*); CC genotype carrier frequency is lower than controls (p=0.006*).
