# Supplementary material for: Analysis of PPARGC1B, RUNX3 and TBKBP1 Polymorphisms in Chinese Han Patients with Ankylosing Spondylitis: A Case-Control Study
Source: PLoS One. 2013 Apr 18;8(4):e61527. doi: 10.1371/journal.pone.0061527 (PMC3630117; doi:10.1371/journal.pone.0061527)
Supplement: Table S2 — Genotype and allele frequencies of RUNX3 SNPs among all AS patients, severe AS patients, normal AS patients versus controls. SNPs in RUNX3 are compared between all AS patients, severe AS patients, and normal AS patients versus the control subjects. # indicates p-value is less than 0.05 but cannot pass Bonferroni correction which shows marginal significant difference. *indicates p-value is less than 0.01 which shows significant difference after Bonferroni correction. After Bonferroni correction, the rs1395621 SNP shows significant difference when comparing severe AS patients to controls, AA genotype carrier frequency is lower than controls (p = 0.007*) and A allele carrier frequency is lower than controls (p = 0.003*). The rs9438876 SNP shows significant difference when comparing normal AS patients to controls, the AA genotype is lower than controls (p = 0.007*). (DOCX) [file pone.0061527.s004.docx]

Table S2. Genotype and allele frequencies of *RUNX3* SNPs among all AS patients, severe AS patients, normal AS patients versus controls.

| SNP |  | All AS subjects cases / controls | |  | Severe AS subjects cases / controls | |  | Normal AS subjects cases / controls | |  |
| --- | --- | --- | --- | --- | --- | --- | --- | --- | --- | --- |
|  |  | frequencies | OR(95% CI) | p | frequencies | OR(95% CI) | p | frequencies | OR(95% CI) | p |
| **rs11249215** | All |  |  | 0.442 |  |  | 0.092 |  |  | 0.448 |
| Genotype | GG | 64/54 | 1.351(0.881~2.073) |  | 18/54 | 1.551(0.801~3.003) |  | 46/54 | 0.902(0.588~1.385) |  |
|  | AG | 186/186 | 1.133(0.836~1.536) |  | 30/186 | 0.826(0.481~1.421) |  | 156/186 | 0.894(0.620~1.288) |  |
|  | AA | 146/162 | 1 |  | 34/162 | 1 |  | 112/162 | 1 |  |
| Allele | G | 314/294 | 1.140(0.931~1.395) |  | 66/294 | 1.168(0.829~1.647) |  | 248/294 | 1.132(0.913~1.404) |  |
|  | A | 478/510 | 1 |  | 98/510 | 1 |  | 380/510 | 1 |  |
|  |  |  |  |  |  |  |  |  |  |  |
| **rs7551188** | All |  |  | 0.361 |  |  | 0.100 |  |  | 0.696 |
| Genotype | TT | 86/94 | 0.793(0.530~1.186) |  | 14/94 | 0.508(0.251~1.031) |  | 72/94 | 0.878(0.573~1.345) |  |
|  | CT | 200/214 | 0.809(0.576~1.137) |  | 40/214 | 0.583(0.336~1.011) |  | 160/214 | 0.857(0.598~1.229) |  |
|  | CC | 110/94 | 1 |  | 28/94 | 1 |  | 82/94 | 1 |  |
| Allele | T | 372/402 | 0.886(0728~1.078) |  | 68/402 | 0.708(0.504~0.995) | **0.046#** | 304/402 | 0.938(0.761~1.156) |  |
|  | C | 420/402 | 1 |  | 96/402 | 1 |  | 324/402 | 1 |  |
|  |  |  |  |  |  |  |  |  |  |  |
| **rs1395621** | All |  |  | **0.041#** |  |  | **0.008*** |  |  | 0.147 |
| Genotype | AA | 58/66 | 0.730(0.477~1.117) |  | 6/66 | 0.408(0.178~0.934) | **0.007*** | 52/66 | 0.834(0.534~1.303) |  |
|  | AG | 189/220 | 0.689(0.504~0.943) | **0.015#** | 39/220 | 0.538(0.321~0.903) | **0.025#** | 150/220 | 0.736(0.527~1.028) |  |
|  | GG | 149/118 | 1 |  | 37/118 | 1 |  | 112/118 | 1 |  |
| Allele | A | 305/352 | 0.811(0.665~0.991) | **0.040#** | 51/352 | 0.585(0.408~0.837) | **0.003*** | 254/352 | 0.880(0.712~1.087) |  |
|  | G | 487/456 | 1 |  | 113/456 | 1 |  | 374/456 | 1 |  |
|  |  |  |  |  |  |  |  |  |  |  |
| **rs4648884** | All |  |  | 0.318 |  |  | 0.632 |  |  | 0.364 |
| Genotype | CC | 62/64 | 0.850(0.554~1.304) |  | 14/64 | 0.915(0.446~1.878) |  | 48/64 | 0.824(0.521~1.301) |  |
|  | CT | 196/220 | 0.784(0.574~1.071) |  | 40/220 | 0.749(0.438~1.282) |  | 156/220 | 0.786(0.564~1.096) |  |
|  | TT | 136/120 | 1 |  | 28/120 | 1 |  | 108/120 | 1 |  |
| Allele | C | 320/348 | 0.904(0.741~1.103) |  | 68/348 | 0.936(0.666~1.316) |  | 252/348 | 0.895(0.724~1.107) |  |
|  | T | 468/460 | 1 |  | 96/460 | 1 |  | 372/460 | 1 |  |
|  |  |  |  |  |  |  |  |  |  |  |
| **rs9438876** | All |  |  | **0.022#** |  |  | 0.716 |  |  | **0.004*** |
| Genotype | GG | 43/30 | 1.347(0.807~2.251) |  | 4/30 | 0.556(0.180~1.713) |  | 39/30 | 1.590(0.937~2.699) |  |
|  | AG | 129/172 | 0.743(0.547~1.008) | **0.047#** | 32/172 | 0.905(0.538~1.523) |  | 97/172 | 0.701(0.504~0.974) | **0.028#** |
|  | AA | 188/184 | 1 | **0.038#** | 38/184 | 1 |  | 150/184 | 1 | **0.007*** |
| Allele | G | 215/232 | 0.991(0.794~1.237) |  | 40/232 | 0.862(0.581~1.278) |  | 175/232 | 1.026(0.811~1.298) |  |
|  | A | 505/540 | 1 |  | 108/540 | 1 |  | 397/540 | 1 |  |

SNPs in *RUNX3* are compared between all AS patients, severe AS patients, and normal AS patients versus the control subjects. # indicates p-value is less than 0.05 but cannot pass Bonferroni correction which shows marginal significant difference. *indicates p-value is less than 0.01 which shows significant difference after Bonferroni correction. After Bonferroni correction, the rs1395621 SNP shows significant difference when comparing severe AS patients to controls, AA genotype carrier frequency is lower than controls (p=0.007*) and A allele carrier frequency is lower than controls (p=0.003*). The rs9438876 SNP shows significant difference when comparing normal AS patients to controls, the AA genotype is lower than controls (p=0.007*)
