# Supplementary material for: Analysis of PPARGC1B, RUNX3 and TBKBP1 Polymorphisms in Chinese Han Patients with Ankylosing Spondylitis: A Case-Control Study
Source: PLoS One. 2013 Apr 18;8(4):e61527. doi: 10.1371/journal.pone.0061527 (PMC3630117; doi:10.1371/journal.pone.0061527)
Supplement: Table S3 — Genotype and allele frequencies of TBKBP1 SNPs among all AS patients, severe AS patients, normal AS patients versus controls. SNPs in TBKBP1 are compared between all AS patients, severe AS patients, and normal AS patients versus the control subjects. # indicates p-value is less than 0.05 but cannot pass Bonferroni correction which shows marginal significant difference. *indicates p-value shows significant difference after Bonferroni correction. After Bonferroni correction the rs8070463 SNP shows significant difference in genotype distribution when comparing severe AS patients to controls, CC genotype carrier frequency is lower than controls (p = 0.003*). This SNP also shows significant difference in C allele distribution when comparing severe AS patients to controls, C allele carrier frequency is lower than controls (p = 0.004*). The rs8070463 SNP is related to the severity of AS. (DOCX) [file pone.0061527.s005.docx]

Table S3. Genotype and allele frequencies of *TBKBP1* SNPs among all AS patients, severe AS patients, normal AS patients versus controls.

| SNP |  | All AS subjects cases / controls | |  | Severe AS subjects cases / controls | |  | Normal AS subjects cases / controls | |  |
| --- | --- | --- | --- | --- | --- | --- | --- | --- | --- | --- |
|  |  | frequencies | OR(95% CI) | p | frequencies | OR(95% CI) | p | frequencies | OR(95% CI) | p |
| **rs8070463** | All |  |  | 0.187 |  |  | **0.014#** |  |  | 0.415 |
| Genotype | CC | 72/94 | 0.788(0.531~1.170) |  | 8/94 | 0.400(0.186~0.861) | **0.003*** | 64/94 | 0.928(0.611~1.411) |  |
|  | CT | 198/184 | 1.084(0.786~1.494) |  | 40/184 | 0.815(0.483~1.376) |  | 158/184 | 1.170(0.828~1.654) |  |
|  | TT | 124/124 | 1 |  | 34/124 | 1 |  | 90/124 | 1 |  |
| Allele | C | 342/372 | 0.890(0.731~1.085) |  | 56/372 | 0.602(0.424~0.856) | **0.004*** | 286/372 | 0.983(0.797~1.212) |  |
|  | T | 446/432 | 1 |  | 108/432 | 1 |  | 338/432 | 1 |  |
|  |  |  |  |  |  |  |  |  |  |  |
| **rs4439799** | All |  |  | 0.670 |  |  | 0.581 |  |  | 0.431 |
| Genotype | TT | 60/64 | 1.016(0.671~1.541) |  | 10/64 | 0.671(0.314~1.435) |  | 50/64 | 1.139(0.733~1.770) |  |
|  | CT | 182/174 | 1.130(0.834~1.530) |  | 34/174 | 0.813(0.485~1.364) |  | 148/174 | 1.219(0.881~1.687) |  |
|  | CC | 152/166 | 1 |  | 38/166 | 1 |  | 114/166 | 1 |  |
| Allele | T | 302/302 | 1.041(0.850~1.275) |  | 54/302 | 0.823(0.576~1.174) |  | 248/302 | 1.105(0.892~1.370) |  |
|  | C | 486/506 | 1 |  | 110/506 | 1 |  | 376/506 | 1 |  |

SNPs in *TBKBP1* are compared between all AS patients, severe AS patients, and normal AS patients versus the control subjects. # indicates p-value is less than 0.05 but cannot pass Bonferroni correction which shows marginal significant difference. *indicates p-value shows significant difference after Bonferroni correction. After Bonferroni correction the rs8070463 SNP shows significant difference in genotype distribution when comparing severe AS patients to controls, CC genotype carrier frequency is lower than controls (p=0.003*). This SNP also shows significant difference in C allele distribution when comparing severe AS patients to controls, C allele carrier frequency is lower than controls (p=0.004*). The rs8070463 SNP is related to the severity of AS.
